# MODERNIZING EXERCISE WITH TELE-VIDEO TO REACH A RURAL GERIATRIC POPULATION OF VETERANS

**DOI:** 10.1093/geroni/igz038.135

**Published:** 2019-11-08

**Authors:** Stephen C Jennings, Kenneth Manning, Oliver Massey, Janet Prvu Bettger, Candace S Brown, Miriam C Morey

**Affiliations:** 1 Durham VA Geriatric Research, Education, and Clinical Center (GRECC), Veterans Affairs Health Care System, Durham, North Carolina, United States; 2 Durham VA Veterans Affairs Health Care System, Durham, North Carolina, United States; 3 Duke Department of Orthopedics, Duke University Medical Center, Durham, North Carolina, United States; 4 Center for Cognitive Neuroscience, Motivated Cognition and Aging Brain Lab, Duke University, Durham, North Carolina Geriatric Research; Education, and Clinical Center (GRECC), Veterans Affairs Health Care System, Durham, North Carolina, United States; 5 Geriatric Research, Education, and Clinical Center (GRECC), Veterans Affairs Health Care System, Durham, North Carolina Center for the Study of Aging/OAIC, Duke University Medical Center, Durham, North Carolina, United States

## Abstract

Rural Veterans often lack access to health care. Veterans Affairs (VA) supports telehealth technologies to provide services remotely that are comparable to onsite in-person care. We piloted VA Video Connect (VVC), to deliver an interactive exercise program for Veterans modeled on the VA Gerofit Program, a successful facility-based exercise program. VVC connects an exercise physiologist directly to the home with smart devices. Invitations to join Gerofit were mailed to 216 rural Veterans. Of 17 respondents, 7 (mean age 68) agreed to VVC tele-exercise 1x week for 12 weeks. Two Veterans were lost to follow-up prior to enrollment. Baseline VVC assessments (N=5) were indicative of high functional impairment in comparison to age-based norms: 2-minute step test (67.2 steps, 5th%tile), 30-second chair stands (12.4 stands, 26th%tile), and 30-second arm curls (15.3 curls, 25th%tile). Feasibility, barriers, and program impact will be discussed. Functional impairment indicates need for telehealth to reach Rural Veterans.

